# Efficient Subtractive Cloning of Genes Activated by Lipopolysaccharide and Interferon γ in Primary-Cultured Cortical Cells of Newborn Mice

**DOI:** 10.1371/journal.pone.0079236

**Published:** 2013-11-11

**Authors:** Osamu Miyauchi, Katsuro Iwase, Kanako Itoh, Masaki Kato, Naohiko Seki, Olivier Braissant, Claude Bachmann, Makio Shozu, Souei Sekiya, Hisao Osada, Masaki Takiguchi

**Affiliations:** 1 Department of Biochemistry and Genetics, Chiba University Graduate School of Medicine, Chiba, Japan; 2 Department of Reproductive Medicine, Chiba University Graduate School of Medicine, Chiba, Japan; 3 Department of Functional Genomics, Chiba University Graduate School of Medicine, Chiba, Japan; 4 Laboratoire Central de Chimie Clinique, Centre Hospitalier Universitaire Vaudois, Lausanne, Switzerland; University of Cologne, Germany

## Abstract

Innate immune responses play a central role in neuroprotection and neurotoxicity during inflammatory processes that are triggered by pathogen-associated molecular pattern-exhibiting agents such as bacterial lipopolysaccharide (LPS) and that are modulated by inflammatory cytokines such as interferon γ (IFNγ). Recent findings describing the unexpected complexity of mammalian genomes and transcriptomes have stimulated further identification of novel transcripts involved in specific physiological and pathological processes, such as the neural innate immune response that alters the expression of many genes. We developed a system for efficient subtractive cloning that employs both sense and antisense cRNA drivers, and coupled it with in-house cDNA microarray analysis. This system enabled effective direct cloning of differentially expressed transcripts, from a small amount (0.5 µg) of total RNA. We applied this system to isolation of genes activated by LPS and IFNγ in primary-cultured cortical cells that were derived from newborn mice, to investigate the mechanisms involved in neuroprotection and neurotoxicity in maternal/perinatal infections that cause various brain injuries including periventricular leukomalacia. A number of genes involved in the immune and inflammatory response were identified, showing that neonatal neuronal/glial cells are highly responsive to LPS and IFNγ. Subsequent RNA blot analysis revealed that the identified genes were activated by LPS and IFNγ in a cooperative or distinctive manner, thereby supporting the notion that these bacterial and cellular inflammatory mediators can affect the brain through direct but complicated pathways. We also identified several novel clones of apparently non-coding RNAs that potentially harbor various regulatory functions. Characterization of the presently identified genes will give insights into mechanisms and interventions not only for perinatal infection-induced brain damage, but also for many other innate immunity-related brain disorders.

## Introduction

Innate immune responses are pivotal in neuroprotection and neurotoxicity during various inflammatory processes, which are triggered by agents such as bacterial lipopolysaccharide (LPS) and modulated by inflammatory cytokines including interferon γ (IFNγ) produced mainly by T cells and natural killer cells [1]–[3]. For example, LPS and IFNγ are thought to be involved in brain disorders [4]–[6] such as those arising from perinatal intrauterine infections that cause various brain injuries ranging from periventricular leukomalacia (PVL) with permanent motor impairment [4], [7] to adult-onset neuropsychiatric disorders [8], [9]. In the central nervous system (CNS), pathogen-associated molecular patterns of infectant products, including LPS, peptidoglycans, and nucleic acids, are recognized by pattern recognition receptors, i.e. Toll-like receptor (TLR) family members. Almost all TLR family members exist on microglia, and some exist on astrocytes, oligodendrocytes, and neurons [2], [10], [11]. Some TLRs can also recognize endogenous cell damage-derived substances, and subsequently activate the pathways that lead to noninfectious disorders, such as traumas, ischemia, autoimmune diseases, and neurodegenerative disorders of the CNS.

Microglia and astrocytes are highly responsive to IFNγ, and microglia also to LPS [10], [12], [13], leading to the production of the well-characterized immediate antimicrobial and neurotoxic agent nitric oxide (NO) by NO synthase (NOS) isoforms such as inducible NOS (iNOS) and endothelial NOS (eNOS) [14]–[19]. In addition to the NO system, comprehensive gene expression analyses, primarily using microarrays, have identified many other candidate genes involved in the LPS and IFNγ responses [20]–[25]. These studies have led to recent characterizations of injury type-specific markers [26] and inflammatory signaling pathways [27] in astrocytes, and action mechanisms of immunosuppressive agents in microglia [28].

The recent characterization of mammalian genomes and transcriptomes harboring unexpected complexity has prompted us to further isolate novel transcripts involved in specific physiological and pathological processes such as neural innate immunity. Besides microarray analysis [29], the identification of differentially expressed genes has been accomplished by subtractive cloning (e.g. [30], [31]) differential display [32], serial analysis of gene expression [33], and next-generation sequencing [34]. Among these procedures, subtractive cloning is the most direct method to isolate novel cDNA clones, and its refinement can fulfill current requests to provide specific transcriptome resources. We recently developed a system that is suitable for both the construction of cDNA libraries and the quantification of mRNA levels from only a small amount of mRNA [35], [36]. Here, we applied this system to subtractive cloning. Specifically, efficient subtraction was accomplished by using the amplified cDNA as a tester and the mixture of both strands of cRNAs as a driver. The subtracted cDNA served for preparation and analysis of microarrays, thereby providing a highly efficient system for isolation of differentially expressed genes. We employed this new system to identify genes activated by LPS and IFNγ in primary-cultured neuronal/glial cells derived from newborn mice as a means to study the pathophysiology of PVL, while the genes identified in this study may be also involved in a broad spectrum of other brain injuries related to innate immunity.

## Materials and Methods

### Animals

Pregnant C57BL/6 mice were purchased from Nippon Clea Co. (Tokyo, Japan), and housed at 24±1°C on a 12-h light/12-h dark cycle with free access to food and water in the Laboratory Animal Center of Chiba University School of Medicine. All experimental procedures in this study were approved by the Animal Experiment Committee of Chiba University (Permit Numbers: 980005 and 20000008), and were conducted in accordance with the Guidelines for Proper Conduct of Animal Experiments of the Science Council of Japan.

### Cell Culture

The neonatal mouse neuronal/glial mixed culture was essentially performed as previously described [37]. The cerebral cortices were removed at postnatal day 1 and placed in the Eagle’s minimum essential medium (Nissui Pharmaceutical Co., Tokyo, Japan)/Hank’s balanced salt solution (1∶1) mixture containing 10 mM l-glutamine (Invitrogen Corp., Carlsbad, CA), 0.2% NaHCO_3_, 10% fetal bovine serum (Invitrogen), 10% horse serum (Invitrogen), 2% Nu serum (Becton Dickinson Labware, Bedford, MA), and 12 ng/mL of nerve growth factor (Sigma Chemical Co., St. Louis, MO). Cells were mechanically dissociated by trituration using fire-polished Pasteur pipettes and seeded in 100-mm dishes coated with 0.2% polyethyleneimine (Sigma). The cultured cells were maintained in the above mixture without the fetal bovine serum at 37°C in a humidified incubator with 5% CO_2_ atmosphere for 1 week. The cells were stimulated by using 10 µg/mL LPS (*Escherichia coli* serotype O127:B8; Sigma) and 500 units/mL IFNγ (recombinant mouse; PBL Biomedical Laboratories, Piscataway, NJ) twice at 6 and 24 h before harvesting for the subtraction experiments.

### cDNA Synthesis and PCR Amplification

**Figure 1:**
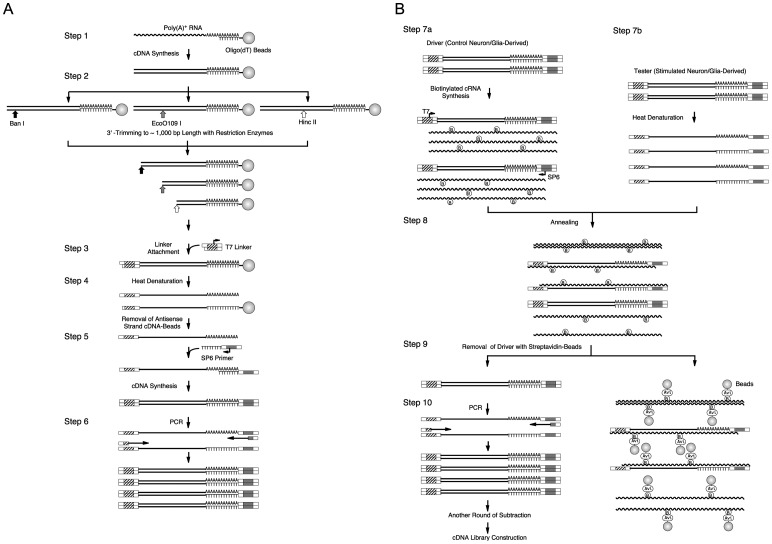
Schematic illustration of cDNA amplification (A) and subsequent subtraction (B). See the text for explanation.

Total RNA was prepared from the stimulated (LPS and IFNγ) and non-stimulated primary-cultured neuronal/glial cells using the acid-guanidine-phenol-chloroform method [38], and was processed to amplify the total cDNA using PCR as schematically illustrated in Fig. 1A. The detailed procedures for Steps 1 and 3–6 were previously described [36]. Briefly, poly(A)^+^ RNA derived from 0.5 µg of total RNA was absorbed onto 50 µg of oligo(dT) magnetic beads Dynabeads Oligo(dT)_25_ (Dynal, Oslo, Norway), and was subjected to first-strand and second-strand cDNA synthesis (Fig. 1A, Step 1). Trimming of the resultant double-stranded cDNA to an estimated average of 1,024 bp lengths from the 3′-termini (Step 2) was performed by dividing the bead-fixed cDNA into three aliquots, each of which was digested with one of three restriction enzymes [*Ban*I, *Eco*O109I, and *Hin*cII (0.5 units)] in 20 µL of the buffer solution consisting of 20 mM Tris-HCl (pH 7.9), 10 mM MgCl_2,_ 50 mM KCl, and 1 mM DTT at 37°C for 1 h. The reaction was stopped by adding 0.8 µL of 0.5 M EDTA-Na (pH 8.0), and the enzymes were inactivated by heating the mixture at 65°C for 20 min. The three 3′-trimmed cDNA-bead suspensions were combined, and the beads were washed three times with 50 µL of 10 mM Tris-HCl (pH 8.0)/1 mM EDTA (Tris-EDTA buffer, TE). The bead-fixed 3′-trimmed cDNA was blunt-ended with 0.5 units of T4 DNA polymerase (Roche Diagnostics, Tokyo, Japan) in 20 µL of a mixture containing 50 mM Tris·HCl (pH 8.8), 15 mM (NH_4_)_2_SO_4_, 7 mM MgCl_2_, 0.1 mM EDTA, 10 mM 2-mercaptoethanol, 0.02 mg/mL bovine serum albumin, and 0.1 mM each of dATP, dCTP, dGTP, and dTTP at 16°C for 10 min. The reaction was stopped by adding 0.8 µL of 0.5 M EDTA-Na (pH 8.0), and the beads were washed three times with 50 µL of TE. The subsequent reactions were performed essentially as previously described [36]. The bead-fixed blunt-ended cDNA was ligated with a linker containing the T7 promoter sequence (Step 3). The sense-strand cDNA was liberated by heat-denaturation (Step 4), and was again converted to the double-stranded form using the oligo(dT) primer containing the SP6 promoter sequence (Step 5). The double-stranded cDNA was amplified by PCR using known sequences at both ends as primers (Step 6).

### Subtraction Procedures

Driver cRNAs were prepared using the amplified total cDNA derived from the non-stimulated control cells as a template (Fig. 1B, Step 7a). The sense and antisense strand cRNAs were specifically synthesized using T7 and SP6 RNA polymerases, respectively. The reaction was performed in 20 µL of a mixture containing 40 mM Tris-HCl (pH 8.0), 6 mM MgCl_2_, 10 mM DTT, 2 mM spermidine, 1 mM ATP, 1 mM CTP, 1 mM GTP, 0.9 mM UTP, 0.1 mM biotin-16-UTP (Roche Diagnostics, Mannheim, Germany), 20 units of human placental RNase inhibitor (Toyobo, Osaka, Japan), 0.3 µg of the amplified total cDNA, and 40 units of T7 or SP6 RNA polymerase (Roche Diagnostics) at 37°C for 2 h. Then, 20 units of DNase I (Roche Diagnostics) were added to digest the template cDNA, and the mixture was further incubated for 15 min. The synthesized cRNA was recovered by ethanol precipitation, washed with 70% ethanol, and dissolved in 20 µL of H_2_O.

Tester cDNA (0.5 µg) derived from the LPS/IFNγ-stimulated cells was mixed with 5 µg of the driver biotinylated cRNAs (2.5 µg each of sense and antisense strands) and dissolved in 30 µL of H_2_O. To avoid hybridization at the linker sequences of both ends, 2 µg of the upper strand of the 5′-T7 linker and 2 µg of the 3′-SP6 oligo(dT) primer were added. The samples were made up to 0.3 M sodium acetate, precipitated with two volumes of ethanol, washed with 70% ethanol, and dissolved in 5 µL of 50 mM Hepes-Na (pH 8.3), 0.5 M NaCl, 0.02 mM EDTA, and 30% formamide. After heat denaturation at 98°C for 90 s to dissociate the tester double-strand cDNA (Step 7b), hybridization was performed at 68°C for 21 h (Step 8).

To remove the biotinylated cRNA-containing mixtures (Step 9), 1 mg of streptavidin-magnetic-beads Dynabeads M-280 Streptavidin (Dynal) in 45 µL of H_2_O plus 50 µL of the buffer solution containing 10 mM Tris-HCl (pH 8.0), 1 mM EDTA, and 2 M NaCl was added to the hybridized mixtures. The binding reaction of biotin and avidin was performed at room temperature for 10 min. After incubation at 55°C for 3 min to release the non-specific binding, the streptavidin beads harboring biotinylated cRNA-containing mixtures were absorbed using magnets, and the subtracted cDNA in the solution was recovered. After the addition of 20 µg of glycogen as a carrier, the subtracted cDNA was ethanol-precipitated, washed with 70% ethanol, and dissolved in 20 µL of TE.

To amplify the subtracted cDNA (Step 10), PCR was performed in eight tubes each containing 50 µL of a mixture consisting of 20 mM Tris-HCl (pH 8.8), 10 mM KCl, 10 mM (NH_4_)_2_SO_4_, 2 mM Mg SO_4_, 0.1% Triton X-100, 0.2 mM each of dATP, dCTP, dGTP, and dTTP, 0.1 µg/µL bovine serum albumin, 1 µL of the subtracted cDNA solution, 50 pmol each of the 5′ and 3′ PCR primers, and 1.8 units of PfuTurbo DNA polymerase (Stratagene, La Jolla, CA). After heat-treatment at 94°C for 3 min, amplification was performed using 13 cycles of 1 min at 94°C, 2 min at 64°C, and 4 min at 72°C. The eight reactions were combined and supplemented with 20 µg of glycogen as a carrier. After extraction with phenol twice, phenol/chloroform twice, and chloroform twice, the PCR products were recovered by ethanol precipitation, washed with 70% ethanol, and dissolved in 20 µL of TE. Typically, 2 µg of PCR products from the subtracted cDNA were obtained. Using 0.5 µg of the subtracted cDNA as a tester, subtractive hybridization and PCR amplification were repeated.

Restriction ends for *Ava*I and *Acc*I were constructed on the 5′- and 3′-termini of the amplified cDNA, respectively, by T4 DNA polymerase in the presence of dATP and dTTP, but not dCTP and dGTP, thereby allowing the unidirectional insertion of cDNA into the plasmid vectors [36]. cDNA fragments longer than 600 bp were gel-purified, and ligated with *Ava*I/*Acc*I-cut pUC19. The *Escherichia coli* strain DH10B (Invitrogen) was then transformed using the ligation products.

### cDNA Microarray Analysis

A microarray chip was prepared as previously described [39] using cDNA clones derived from the control cells, LPS/IFNγ-stimulated cells, and subtracted products. Sense-strand cRNA transcribed from the T7 promoter of the bead-fixed cDNA (Fig. 1A, Step 3 without prior restriction enzyme digestion) was subjected to aminoallyl incorporation into cDNA during reverse transcription. The synthesized sense-strand cRNA (0.5 µg) and 2 µg of oligo(dT) in 15.5 µL of solution were heat-denatured at 70°C for 10 min, and immediately cooled on ice. The solution was made up to 30 µL of a mixture containing 50 mM Tris-HCl (pH 8.3), 75 mM KCl, 3 mM MgCl_2_, 10 mM DTT, 0.5 mM each of dATP, dCTP, and dGTP, 0.2 mM 5-(3-aminoallyl)-dUTP (Ambion Inc., Austin, TX), and 400 units of reverse transcriptase SuperScript II (Invitrogen). The reaction was allowed to proceed at 42°C for 60 min. After alkaline degradation of the template RNA, the aminoallyl-modified cDNA was purified using MinElute (Qiagen, Hilden, Germany). Coupling of the aminoallyl-modified cDNA (0.5 µg) with Cy3 or Cy5 CyDye (Amersham Biosciences, Tokyo, Japan) was performed according to the manufacturer’s protocol. The Cy3- and Cy5-labeled cDNAs were mixed, and made up to 40 µL of a solution containing 1.25 µg/µL yeast RNA, 1.25 µg/µL poly(A), 3.4×SSC (1×SSC: 0.15 M NaCl/15 mM sodium citrate), and 0.3% SDS. Hybridization with the microarray was performed at 65°C overnight under humidified conditions. After the hybridization, the array was washed twice for 5 min with 2×SSC/0.1% SDS at room temperature, twice for 5 min with 0.2×SSC/0.1% SDS at 40°C, and finally rinsed with 0.2×SSC. The array was centrifuged at 1,000 rpm for 1 min, and then scanned using the fluorescence laser-scanning device ScanArray4000 (GSI Lumonics, Bedford, MA).

### Southern and Northern Analysis

Amplified total cDNA mixtures (0.2 µg per lane) were separated by 1% agarose gel electrophoresis. DNAs were visualized by ethidium bromide staining, alkaline-denatured, neutralized, and transferred to nylon membranes. Total RNAs (0.5 µg per lane) were electrophoresed in denaturing formaldehyde-agarose (1%) gels. RNAs were visualized by ethidium bromide staining, and blotted onto nylon membranes. For preparation of antisense strand-specific probes, plasmids harboring cloned cDNAs were linearized with an appropriate restriction enzyme, and then subjected to the synthesis of digoxigenin-labeled cRNA probes with SP6 polymerase, using a transcription kit (Roche Diagnostics). Hybridization, washing, and chemiluminescent detection on X-ray films were done as recommended by Roche Diagnostics. Densitometry was performed using ImageJ 1.46r (National Institute of Health, Bethesda, MA).

### Statistical Analysis

The statistical significance of the enrichment of LPS/IFNγ-induced clones by the subtraction was assessed using a Fisher’s exact test.

## Results

### Subtractive Cloning of Genes Activated by LPS and IFNγ

To isolate candidate genes involved in brain injuries such as PVL that is caused by perinatal infections, we used a primary mixed neuronal/glial culture derived from the cerebral cortex of postnatal day 1 mice. At this time point, the culture is expected to resemble the perinatal state of the brain. In addition, the mixed neuronal/glial culture more closely simulates physiological brain conditions than cell-type-specific cultures, and thus provides an excellent starting point for systematic investigations of the neural responses to LPS and IFNγ. The neuronal/glial cells were stimulated with 10 µg/mL LPS and 500 units/mL IFNγ. The LPS dose was chosen based on our previous studies, in which we examined NOS mRNA induction in the rat astroglioma C6 cell line [40] and in the rat brain *in vivo* [18]. The IFNγ dose was chosen based on other previous reports [41], [42], in which its effects on the growth and apoptosis of rat neural progenitor cells were examined.

To enrich the genes activated by LPS and IFNγ in the neuronal/glial cultured cells, we employed the subtractive cloning procedure described in the Materials and Methods and illustrated in Fig. 1. Since only limited amounts of RNA were obtained from the primary-cultured cells, we first amplified the total cDNA by PCR (Fig. 1A). The procedures for Steps 1 and 3–6 [36], and Step 2 in brief [35] have been previously described. Poly(A)^+^ RNA derived from 0.5 µg of total RNA of the stimulated (LPS and IFNγ) or non-stimulated cells was absorbed onto oligo(dT) magnetic beads, and was used as a template for cDNA synthesis (Fig. 1A, Step 1). To avoid the loss of long cDNA during PCR, we performed 3′-trimming of cDNA by digestion using one of three restriction enzymes *Ban*I, *Eco*O109I, and *Hin*cII (Step 2) prior to PCR. The restriction sites for each of these enzymes appear once in an average of 1,024 bp in random DNA sequences, and the combination of separate digestion with the three enzymes allows 99.7% of the cDNA population to be trimmed from the 3′ termini into less than 2,000 bp lengths. The restriction ends were blunted and ligated with a linker harboring the T7 promoter sequence (Step 3). The sense-strand cDNA liberated by heat denaturation (Step 4) was subjected to antisense-strand cDNA synthesis-coupled construction of the SP6 promoter sequence onto the 3′ terminus (Step 5). The total cDNA was amplified by PCR, using the linker sequences of both ends as primers (Step 6).

In the subtractive hybridization (Fig. 1B), we used a mixture of both sense and antisense strand cRNAs as a driver, rather than cDNA. This enabled us to avoid amplification of the contaminating driver during the PCR amplification of subtracted tester cDNA after removal of the driver cRNA complexes. As shown in Fig. 1B, Step 7a, the amplified total cDNA derived from the non-stimulated control cells were subjected to preparation of biotinylated-cRNA drivers, by synthesizing sense and antisense strand cRNAs with T7 and SP6 RNA polymerases, respectively. The tester cDNA derived from the LPS/IFNγ-stimulated cells was mixed with the driver biotinylated-cRNAs, heat-denatured (Step 7b), and allowed to hybridize (Step 8). The resultant subtracted cDNA was separated by removing the biotinylated-cRNA-containing constituents using streptavidin-magnetic-beads (Step 9), and amplified by PCR (Step 10). A portion of the subtracted cDNA was processed, as a tester, to another round of subtractive hybridization and PCR amplification (repetition from Step 7b to Step 10).

**Figure 2:**
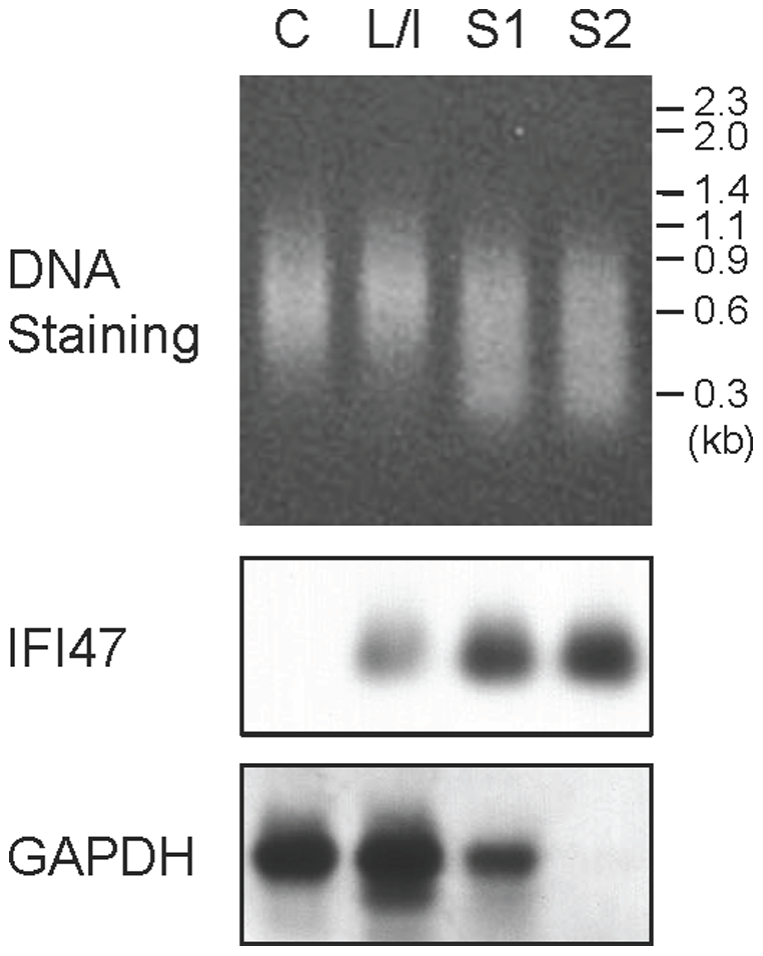
Monitoring subtraction processes. PCR-amplified cDNA mixtures (0.2 µg) derived from the control cells (C), cells stimulated by LPS and IFNγ (L/I), first-round subtracted products (S1), and second-round subtracted products (S2) were electrophoresed, stained (top panel), and subjected to Southern analysis to detect the cDNAs of interferon gamma inducible protein 47 (IFI47) and glyceraldehyde-3-phosphate dehydrogenase (GAPDH).

As shown in Fig. 2, the original total cDNA and subtracted cDNAs were subjected to Southern analysis to determine the subtraction efficiency. Serial two-round subtractions allowed enrichment of cDNA of interferon gamma inducible protein 47 (IFI47) [43], [44] as a positive control. Comparatively, the glyceraldehyde-3-phosphate dehydrogenase (GAPDH) cDNA almost completely vanished after the second-round subtraction. Therefore, the present procedure facilitated effective subtraction.

**Figure 3:**
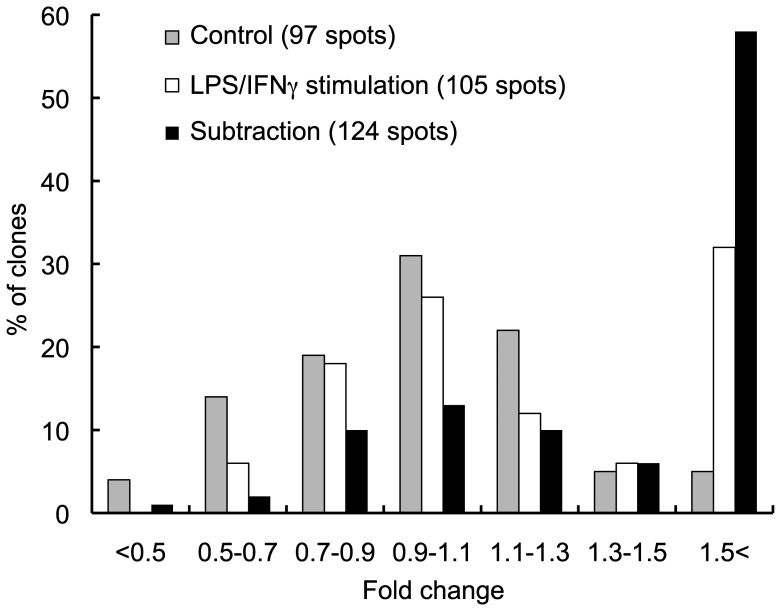
Subtraction efficiency evaluated by cDNA microarray analysis. An in-house microarray containing cDNA clones derived from the control cells (gray bars, 97 spots in total), LPS/IFNγ-stimulated cells (white bars, 105 spots), and subtracted products (black bars, 124 spots) was prepared, and analyzed for changes in the mRNA levels in response to LPS and IFNγ. The percentages of the clones that had altered mRNA levels indicated in the horizontal axis are plotted for each clone group.

Our system enabled the construction of unique restriction ends on 5′ and 3′ termini of amplified cDNA fragments [36], and then their unidirectional insertion into plasmids. We prepared a library of the subtracted cDNA, as well as of control cell-derived and LPS/IFNγ-stimulated cell-derived cDNA, and determined the 5′-end sequences of randomly-selected cDNA clones. We then constructed an in-house microarray containing 124 subtracted cDNA clones, as well as 97 control cell-derived and 105 LPS/IFNγ-stimulated cell-derived cDNA clones. The array was hybridized with a target mixture of Cy3-labeled cDNA derived from LPS/IFNγ-stimulated cells and Cy5-labeled cDNA derived from non-stimulated control cells. Hybridization was also performed with a target mixture of cDNAs interchanged for Cy3/Cy5 labeling to correct possible biases such as intensity-dependent fluorescence differences between the two dyes. The results of the two hybridizations were averaged, and the distribution of fold-changes in the mRNA levels in response to LPS/IFNγ stimulation was determined for the three groups of array spots: control cell-derived, LPS/IFNγ-stimulated cell-derived, and subtracted cDNAs (Fig. 3). Importantly, the subtracted cDNA population was enriched with LPS/IFNγ-activated genes. Indeed, 58% of the subtracted clones had at least 1.5-fold change. This distribution is significantly (*P*<0.001, Fisher’s exact test) higher than that of the clones derived from the LPS/IFNγ-stimulated cells, thereby confirming the successful enrichment of the LPS/IFNγ-induced clones by subtraction.

### Predominant Categories of Genes Activated by LPS and IFNγ

The LPS/IFNγ-activated genes with at least 1.5-fold change in the array analysis are listed in Table 1 after eliminating the redundancies. Strikingly, genes belonging to three categories were predominant. The first category includes chemokine genes: chemokine (C-C motif) ligand 2 (*Ccl2*; No. 8), *Ccl5* (No. 1), *Ccl7* (No. 5), *Ccl19* (No. 45), chemokine (C-X-C motif) ligand 1 (*Cxcl1*; No. 31), *Cxcl9* (No. 4), *Cxcl10* (No. 11), and *Cxcl11* (No. 14). The second contains genes for GTPases involved in protection against intracellular pathogens [44]–[46]: guanylate binding protein 2 (*Gbp2*; No. 10), *Gbp3* (No. 40), *Gbp4* (No. 20), *Gbp6* (No. 7), *Ifi47* (No. 16), immunity-related GTPase family M member 2 (*Irgm2*; No. 23), and T-cell specific GTPase 2 (*Tgtp2*; No. 3). The third category pertains to genes involved in antigen presentation: histocompatibility 2 (H2), class II antigen A, β1 (*H2-Ab1*; No. 26), *H2-DMa* (No. 25), *H2-K1* (No. 22), *H2-Q10* (No. 58), β_2_ microglobulin (*B2m*; No. 37), proteasome subunit β8 (*Psmb8*; No. 27), *Psmb9* (No. 18), *Psmb10* (No. 32), transporter associated with antigen processing 1 (*Tap1*; No. 21); and possibly proteasome assembly chaperone 4 (*Psmg4*; No. 52), ubiquitin D (*Ubd*; No. 13), and ubiquitin-conjugating enzyme E2M (*Ube2m*; No. 60).

**Table 1:** Genes activated by LPS and IFNγ.

No.	Foldinduction	Subtrac-tion^a^	Accession	Gene name
1	80.12	S	NM_013653.3	Ccl5 chemokine (C-C motif) ligand 5
2	64.10	S	NM_011315.3	Saa3 serum amyloid A 3
3	57.13		NM_001145164.1	Tgtp2 T-cell specific GTPase 2
4	51.70	S	NM_008599.4	Cxcl9 chemokine (C-X-C motif) ligand 9
5	50.91	S	NM_013654.3	Ccl7 chemokine (C-C motif) ligand 7
6	49.94	S	NM_008491.1	Lcn2 lipocalin 2
7	49.87	S	NM_194336.2	Gbp6 guanylate binding protein 6
8	44.82	S	NM_011333.3	Ccl2 chemokine (C-C motif) ligand 2
9	33.02	S	NM_011018.2	Sqstm1 sequestosome 1
10	31.61	S	NM_010260.1	Gbp2 guanylate binding protein 2
11	30.66	S	NM_021274.1	Cxcl10 chemokine (C-X-C motif) ligand 10
12	30.24	S	NM_010738.2	Ly6a lymphocyte antigen 6 complex, locus A
13	28.95	S	NM_023137.3	Ubd ubiquitin D
14	26.36	S	NM_019494.1	Cxcl11 chemokine (C-X-C motif) ligand 11
15	26.11	S	NM_009778.2	C3 complement component 3
16	24.00	S	NM_008330.1	Ifi47 interferon gamma inducible protein 47
17	22.21	S	NM_009396.2	Tnfaip2 tumor necrosis factor, alpha-induced protein 2
18	18.23	S	NM_013585.2	Psmb9 proteasome (prosome, macropain) subunit, beta type 9 (largemultifunctional peptidase 2)
19	17.66	S	NM_144938.2	C1s complement component 1, s subcomponent
20	17.13	S	NM_008620.3	Gbp4 guanylate binding protein 4
21	13.67	S	NM_013683.2	Tap1 transporter 1, ATP-binding cassette, sub-family B (MDR/TAP)
22	13.14	S	NM_001001892.2	H2-K1 histocompatibility 2, K1, K region
23	11.81		NM_019440.3	Irgm2 immunity-related GTPase family M member 2
24	10.36	S	NM_139198.2	Plac8 placenta-specific 8
25	10.31	S	NM_010386.3	H2-DMa histocompatibility 2, class II, locus DMa
26	10.23	S	NM_207105.3	H2-Ab1 histocompatibility 2, class II antigen A, beta 1
27	8.50	S	NM_010724.2	Psmb8 proteasome (prosome, macropain) subunit, beta type 8 (largemultifunctional peptidase 7)
28	7.79	S	NM_008198.2	Cfb complement factor B
29	7.76	S	NM_013671.3	Sod2 superoxide dismutase 2, mitochondrial
30	7.69		NM_008987.3	Ptx3 pentraxin related gene
31	6.67	S	NM_008176.3	Cxcl1 chemokine (C-X-C motif) ligand 1
32	6.51	S	NM_013640.3	Psmb10 proteasome (prosome, macropain) subunit, beta type 10
33	6.48	S	NM_007575.2	Ciita class II transactivator
34	6.44		NM_010493.2	Icam1 intercellular adhesion molecule 1
35	5.80	S	NM_010501.2	Ifit3 interferon-induced protein with tetratricopeptide repeats 3
36	5.78	S	NM_133662.2	Ier3 immediate early response 3
37	5.72		NM_009735.3	B2m beta-2 microglobulin
38	5.40		NM_010579.2	Eif6 eukaryotic translation initiation factor 6
39	5.34		NM_011693.3	Vcam1 vascular cell adhesion molecule 1
40	4.76		NM_018734.3	Gbp3 guanylate binding protein 3
41	4.40		NM_007752.2	Cp ceruloplasmin
42	4.10	S	AB811352	LIs01G08 chr5∶92,787,601-92,787,879 (minus strand)
43	3.94	S	NM_025992.2	Herc6 hect domain and RLD 6
44	3.49	S	NM_019946.4	Mgst1 microsomal glutathione S-transferase 1
45	3.35	S	NM_011888.2	Ccl19 chemokine (C-C motif) ligand 19
46	3.22	S	NM_026055.1	Rpl39 ribosomal protein L39
47	2.98		NM_008300.3	Hspa4 heat shock protein 4
48	2.86	S	NM_009851.2	Cd44 CD44 antigen
49	2.85		NM_011879.2	Ik IK cytokine
50	2.78		NM_001163590.1	Stx11 syntaxin 11
51	2.66	S	NM_008871.2	Serpine1 serine (or cysteine) peptidase inhibitor, clade E, member 1
52	2.62	S	NM_001101430.1	Psmg4 proteasome (prosome, macropain) assembly chaperone 4
53	2.57	S	NM_010681.4	Lama4 laminin, alpha 4
54	2.52	S	NM_145934.1	Stap2 signal transducing adaptor family member 2
55	2.51		NM_133701.2	Prpf6 PRP6 pre-mRNA splicing factor 6 homolog (yeast)
56	2.41	S	NM_175389.4	Rg9mtd2 RNA (guanine-9-) methyltransferase domain containing 2
57	2.39	S	NM_001113529.1	Csf1 colony stimulating factor 1 (macrophage)
58	2.37	S	NM_010391.4	H2-Q10 histocompatibility 2, Q region locus 10
59	2.35	S	NM_021524.2	Nampt nicotinamide phosphoribosyltransferase
60	2.33		NM_145578.2	Ube2m ubiquitin-conjugating enzyme E2M (UBC12 homolog, yeast)
61	2.28		AB811353	Co101C12 chr19∶25,002,842-25,003,439 (plus strand)
62	2.24		NM_021511.2	Rrs1 RRS1 ribosome biogenesis regulator homolog (S. cerevisiae)
63	2.23	S	NM_001083938.2	Rnaset2a ribonuclease T2A
64	2.22		NM_178252.2	Arhgap33 Rho GTPase activating protein 33
65	2.19	S	NM_001008232.2	Asap3 ArfGAP with SH3 domain, ankyrin repeat and PH domain 3
66	2.14	S	NM_018825.3	Sh2b2 SH2B adaptor protein
67	2.14	S	NM_009780.2	C4b complement component 4B (Childo blood group)
68	2.13		NM_001081270.1	Dscaml1 Down syndrome cell adhesion molecule-like 1
69	2.04	S	NM_001205081.1	Trim47 tripartite motif-containing 47
70	1.99		BB142106.1	BB142106 RIKEN full-length enriched, adult female vagina Mus musculus cDNA clone 9930013N19 3-, mRNA sequence
71	1.97	S	NM_011590.2	Timm17a translocase of inner mitochondrial membrane 17a
72	1.95		NM_011937.2	Gnpda1 glucosamine-6-phosphate deaminase 1
73	1.95		NM_028040.2	Rpusd4 RNA pseudouridylate synthase domain containing 4
74	1.95		AB811354	Co301F01 chr17∶17,516,696-17,517,236 (minus strand)
75	1.93	S	NM_010239.1	Fth1 ferritin heavy chain 1
76	1.91		NM_024177.3	Mrpl38 mitochondrial ribosomal protein L38
77	1.89		NM_001165991.1	Arfrp1 ADP-ribosylation factor related protein 1
78	1.82	S	BI990686.1	4074-27 Mouse E14.5 retina lambda ZAP II Library Mus musculus cDNA, mRNA sequence
79	1.81		NM_144804.1	Depdc7 DEP domain containing 7
80	1.78		NM_023142.2	Arpc1b actin related protein 2/3 complex, subunit 1B
81	1.76		NM_001025102.1	2700007P21Rik RIKEN cDNA 2700007P21 gene
82	1.70	S	NM_029166.2	Uhrf1bp1l UHRF1 (ICBP90) binding protein 1-like
83	1.70	S	NM_009294.3	Stx4a syntaxin 4A (placental)
84	1.63		NM_007459.3	Ap2a2 adaptor protein complex AP-2, alpha 2 subunit
85	1.60	S	NM_011034.4	Prdx1 peroxiredoxin 1
86	1.56		NM_177707.3	Stac3 SH3 and cysteine rich domain 3
87	1.54		NM_199304.1	Zfp341 zinc finger protein 341


a Genes obtained by subtractive cloning are marked “S”.

### Differential Gene Activation by LPS and IFNγ

**Figure 4:**
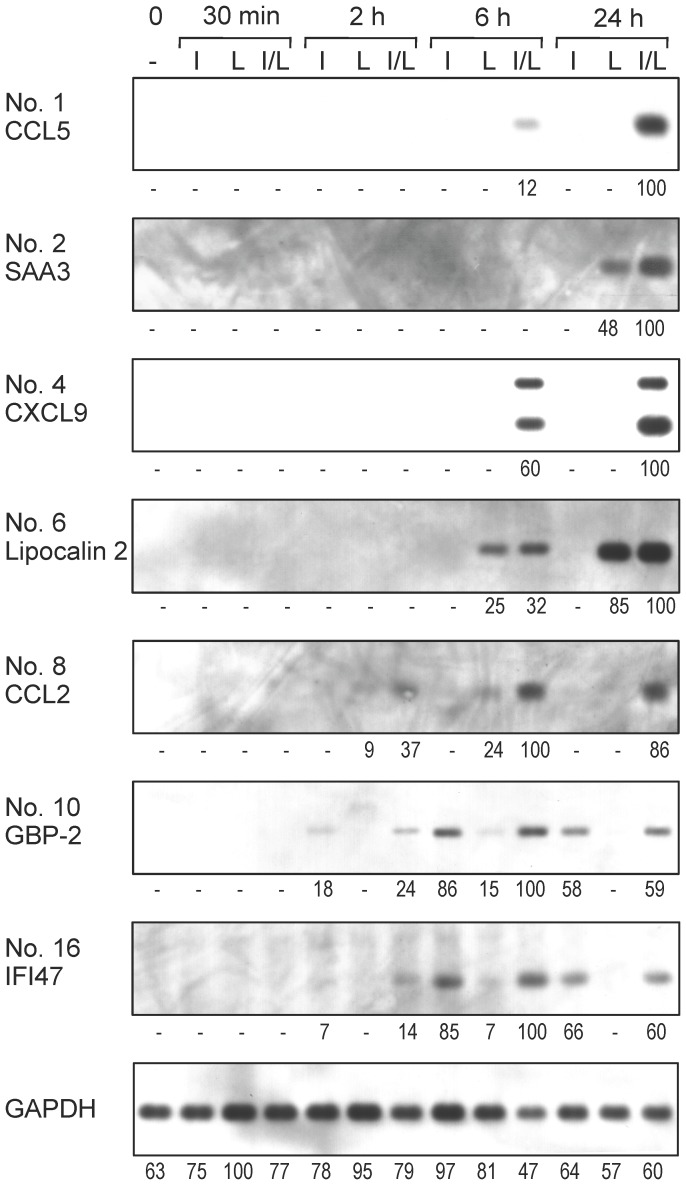
Time course of changes in mRNA levels of several genes in response to LPS and IFNγ. Total RNAs were prepared from primary-cultured neuronal/glial cells stimulated by IFNγ (I), LPS (L), or both (I/L) for indicated periods. RNAs (0.5 µg per lane) were electrophoresed and subjected to Northern analysis for the indicated mRNAs and for GAPDH mRNA as a control. Below the chemiluminogram, the densitometrically quantified band intensities are shown. “−” indicates that the band intensity was below the detectable level.

For several selected genes, the time-dependent changes in the mRNA levels in response to LPS and/or IFNγ were examined by Northern analysis (Fig. 4). In the present experimental system, cDNA clones of the subtracted libraries were ready to serve as templates for antisense strand-specific cRNA probes, as described in the Materials and Methods. Coadministration of LPS and IFNγ was required for strong induction of the mRNAs of the chemokines CCL5 (No. 1), CXCL9 (No. 4), and CCL2 (No. 8). The mRNA levels of serum amyloid A3 (SAA3; No. 2) and lipocalin 2 (No. 6) were mainly responsive to LPS, while those for the GTPases GBP-2 (No. 10) and IFI47 (No. 16) were mainly responsive to IFNγ. Therefore, these genes displayed differential patterns with respect to their responsiveness to LPS and IFNγ. The gene activation profiles in Fig. 4 are concordant with the notion that both LPS and IFNγ can affect the brain cells through direct but complicated means. It remains to be investigated what cell type is responsible for each gene activation in the present mixed neuronal/glial culture system.

### Putative Non-Coding RNAs Induced by LPS and IFNγ

**Figure 5:**
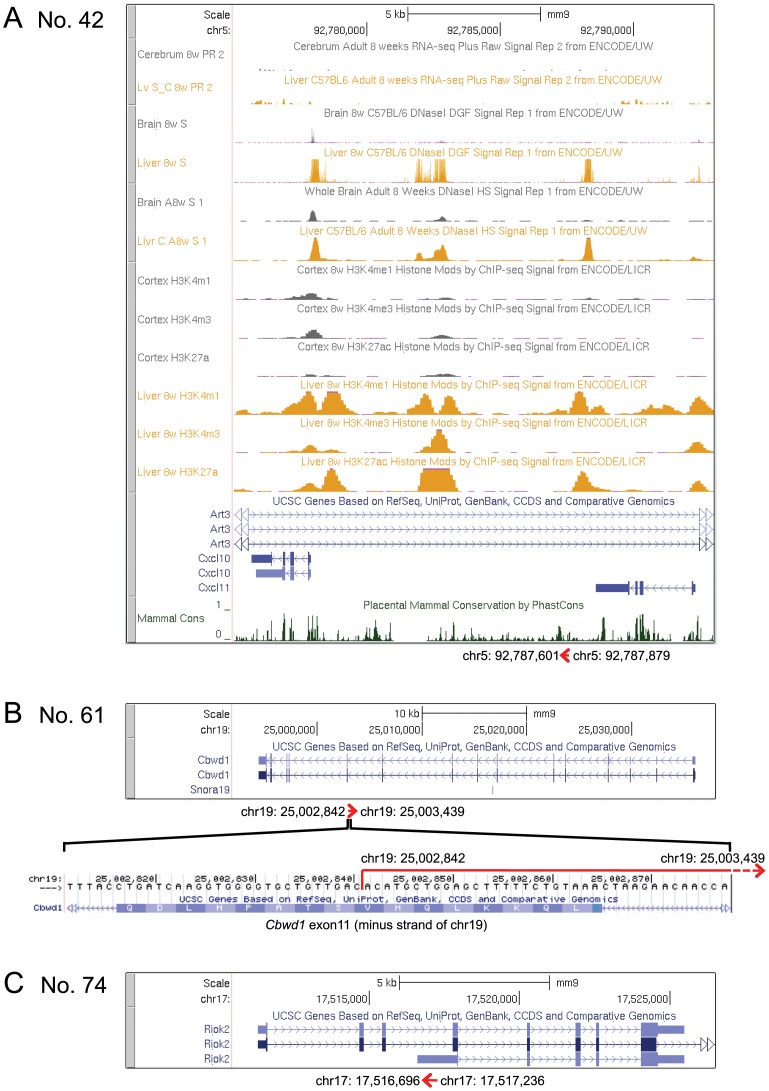
Chromosomal loci of putative non-coding RNAs. The chromosomal loci of the clone Nos. 42 (A), 61 (B), and 74 (C) of Table 1 are shown. The span and direction of the RNAs are shown by red arrows below the screen shots from the UCSC Genome Browser on Mouse July 2007 (NCBI37/mm9) Assembly.

Table 1 includes novel transcripts: clone No. 42 derived from subtracted products, and clone Nos. 61 and 74 derived from control cells. These are apparently non-coding RNAs because even the longest open reading frame harboring the initiation ATG codon corresponded to only 35 amino acids (No. 61, chr19∶25,002,938–25,003,042) in these clones. In Fig. 5, their chromosome loci are represented alongside the screen shots from the University of California Santa Cruz (UCSC) Genome Browser.

Clone No. 42 (Fig. 5A) contained the 279-bp segment corresponding to chr5∶92,787,601–92,787,879 (minus strand) situated between the *Cxcl10* and *Cxcl11* loci, which in turn are located in a large (∼55 kb) intron of *Art3*. As previously noted [20]–[23], [47], [48], *Cxcl10* and *Cxcl11* are activated by LPS and IFNγ (Table 1, Nos. 11 and 14). According to the RNA-Seq data of the UCSC Browser (Fig. 5A), expression levels of *Cxcl10* and *Cxcl11* are low in the normal cerebrum, while higher expression levels are seen in the normal liver that may have been exposed to enteromicrobial products. The locus of clone No. 42 overlaps or adjoins the genome sequences exhibiting the features characteristic of enhancers: sequence conservation in the intergenic regions [49], DNaseI hypersensitivity [50], [51], and binding to histone H3 monomethylated at lysine 4 (H3K4me1) [52] and H3 acetylated at lysine 27 (H3K27ac) [53], [54]. These features are more prominent in the liver than in the brain, which correlates with the expression levels of *Cxcl10* and *Cxcl11*. It is plausible that clone No. 42 corresponds to a transcript started from an enhancer [55], i.e. enhancer RNA (eRNA) [56].

Clone No. 61 (Fig. 5B) contained the 598-bp segment corresponding to chr19∶25,002,842–25,003,439 (plus strand) complementary to the sequence spanning the intron 10–exon 11 junction of the COBW domain containing 1 (*Cbwd1*) gene. This location is reminiscent of roles of antisense RNAs complementary to exon-intron boundaries of mRNA precursors in the regulation of alternative splicing, as has been demonstrated for a number of genes [57]–[59].

Clone No. 74 (Fig. 5C) contained the 541-bp segment corresponding to chr17∶17,516,696–17,517,236 (minus strand) complementary to transcripts of the RIO kinase 2 (*Riok2*) gene. *Riok2* has two major transcription start sites, and the clone No. 74 sequence is complementary to intron 3 or exon 1 of each alternative transcript. It is tempting to speculate that this antisense RNA differentially regulates alternative *Riok2* transcripts in mRNA precursor processing and/or mRNA activity.

## Discussion

Identification of genes with altered expression levels in physiological and pathological processes is a promising strategy to obtain insights into underlying genetic mechanisms. We have developed a system of subtractive cloning coupled with in-house cDNA microarray analysis for the efficient isolation of differentially expressed genes. It enabled the identification of transcripts induced by LPS and IFNγ in primary-cultured mixed neuronal/glial cells derived from neonatal mice. Newly identified genes included those for novel putative non-coding RNAs potentially possessing various regulatory functions, suggesting the feasibility of the present system for direct isolation of the transcripts that represent emerging complexity of mammalian genomes and transcriptomes.

As for protein-coding genes, the list (Table 1) included a number of genes for chemokines, GTPases, and antigen presentation pathways. In previous studies using cultures enriched in microglia [22], [24] and astrocytes [20], [21], [25]–[27] as well as immortalized microglial cell lines [23], [28], these genes have been described as typical examples of genes activated by LPS and IFNγ, which supports the validity of our system to identify differentially expressed genes. Interestingly, a growing number of functions have been assigned to these genes, not only in immunity and inflammation but also in many physiological processes. Chemokines were originally identified as chemotactic cytokines involved in leukocyte migration associated with inflammation [60]. Recently, chemokine receptors have also been found in neurons and glial cells of the CNS [61], and the chemokine system is implicated in CNS functions such as neuromodulatory/neurotransmitter activities [62], [63]. GTPases are best known for their roles in protection against intracellular pathogens [44]–[46]. In addition, GTPases have been assigned functions in processes such as cell proliferation, specification, and death [46]. While genes for antigen presentation pathways are central players in adaptive immunity, their members, such as the major histocompatibility complex class I *H2-Kb and H2-Db* genes, have been recently identified for their roles in the regulation of developmental ocular dominance [64] and adult motor learning [65] as well as neuronal death and motor recovery after ischemia [66]. Therefore, it will be interesting to investigate the roles of the genes encoding chemokines, GTPases, and members of antigen presentation pathways in Table 1 not only as mediators of inflammatory responses but also as regulators of neural development and plasticity in normal and disease-associated processes.

An example of innate immunity-related disorders resulting in severe brain damage is PVL, which is a predominant form of cerebral palsy with white matter injury in preterm infants [67]. PVL is characterized by small multifocal zones of necrosis, in particular around the regions adjacent to the external angles of the lateral ventricles. While hypoxia-ischemia is a likely convergent point leading to PVL, recent clinical and experimental data support a strong correlation between maternal-fetal infection and PVL [4]–[6]. In addition, stimulation of microglial TLR4 by LPS leads to neurodegeneration resembling PVL [68], [69], suggesting the direct causative role of LPS in PVL-associated brain injury. It was proposed that LPS, as well as IFNγ produced during the course of infection, activates microglia and astrocytes, thereby resulting in damaged oligodendrocytes in PVL [4]–[6]. The present study showed that neonatal neuronal/glial cells are highly responsive to LPS and IFNγ and that a large number of genes are activated by these agents. These findings, in addition to the immaturity of the infant blood-brain barrier [70], [71], suggest that LPS and IFNγ during extra-cerebral infections such as intrauterine infection can cause perinatal brain injury in a direct but complicated manner. The mRNA induction profiles in Fig. 4, in which LPS and IFNγ differentially activate target genes, are also concordant with this notion.

Many of the regulatory mechanisms underlying the cooperative or distinctive gene activation by LPS and IFNγ observed in Fig. 4 remain to be investigated. In this study, we used a mixed neuronal/glial culture system, which more closely simulates physiological brain conditions than cell-type-specific cultures. However, use of this system necessitates determining what cell type is responsible for each gene activation. According to previous reports, the LPS receptor TLR4 is expressed on microglia, and is expressed at lower levels, if at all, on astrocytes, oligodendrocytes, and neurons [2; and references therein]. In contrast, the IFNγ receptor is expressed on all of these cell types [72; and references therein]. Previous comprehensive analyses of LPS- and IFNγ-responsive genes in neural cells [20]–[25], [27], [28] were mainly performed using either primary cultures enriched in microglia or astrocytes or established cell lines. Comparison of our results with the results from these previous studies revealed interesting differences. For example, in Fig. 4, strong induction of CCL2, CCL5, and CXCL9 mRNAs required coadministration of LPS and IFNγ, whereas previous studies reported that these mRNAs were induced by administration of LPS alone in primary-cultured microglia [22], astrocytes [25], and microglial BV-2 cells [28], as well as by administration of IFNγ alone in primary-cultured astrocytes [21]. In the present study, strong induction of GBP-2 mRNA mainly depended on IFNγ stimulation (Fig. 4), but, in the previous study [22], it was caused by administration of LPS on primary-cultured microglia. Astrocytes derived from postnatal day 30–35 mice that were administered an intraperitoneal injection of LPS also exhibited strong induction of GBP-2 mRNA [26]. Precise comparative examination of the experimental conditions that caused these differences in the LPS and IFNγ responses may lead to insights into the intercellular regulatory networks among different neuronal/glial cell types.

Besides PVL, TLR4 stimulation is implicated in a number of brain injuries, as well as pathogen elimination [10], [12], [73]. In addition to presumably microbial product-associated diseases such as experimental autoimmune encephalomyelitis, a murine model of multiple sclerosis [74], the involvement of TLR4 has also been reported in apparently noninfectious disorders such as neuropathic pain caused by transection of the spinal nerves [75] and ischemic brain injury [76], [77]. In Alzheimer’s disease, TLR4 may exhibit beneficial or deleterious effects, depending on the conditions of its activation [11]. Recently, the involvement of TLR4 in alcoholism has been reported [78], [79]. Insights into the underlying mechanisms and possible interventions for these TLR4-mediated brain disorders would be also provided by clarifying the roles of the presently identified genes, including those for non-coding RNAs, activated by the natural TLR4 ligand LPS and/or its effective modulator IFNγ.
